# A Review of the Effectiveness of Current US Policies on Antimicrobial Use in Meat and Poultry Production

**DOI:** 10.1007/s40572-022-00351-x

**Published:** 2022-04-27

**Authors:** David Wallinga, Lidwien A. M. Smit, Meghan F. Davis, Joan A. Casey, Keeve E. Nachman

**Affiliations:** 1 Natural Resources Defense Council, San Francisco, CA 94104 USA; 2 Institute for Risk Assessment Sciences (IRAS), Utrecht University, Utrecht, The Netherlands; 3 Department of Environmental Health & Engineering, Johns Hopkins Bloomberg School of Public Health, 615 North Wolfe Street, Room W-7007, Baltimore, MD 21205 USA; 4 Department of Molecular and Comparative Pathobiology, Johns Hopkins School of Medicine, Baltimore, MD 21205 USA; 5 Division of Infectious Diseases, Johns Hopkins School of Medicine, Baltimore, MD 21205 USA; 6 Department of Environmental Health Sciences, Columbia University Mailman School of Public Health, New York, NY 10034 USA; 7 Department of Health Policy and Management, Johns Hopkins Bloomberg School of Public Health, Baltimore, MD 21205 USA; 8 Johns Hopkins Center for a Livable Future, Johns Hopkins Bloomberg School of Public Health, Baltimore, MD 21202 USA; 9 Risk Sciences and Public Policy Institute, Johns Hopkins Bloomberg School of Public Health, Baltimore, MD 21205 USA

**Keywords:** Drug resistance, Microbial, Antimicrobial use, Food animal production, Livestock, Policy, Antibiotic resistance, One Health

## Abstract

**Purpose:**

Industrial food animal production accounts for most animal-source protein consumed in the USA. These operations rely on an array of external inputs, which can include antimicrobials of medical importance. The use of these drugs in this context has been the subject of public health debate for decades because their widespread use contributes to the selection for and proliferation of drug-resistant bacteria and their genetic determinants. Here, we describe legislative and regulatory efforts, at different levels of governance in the USA, to curtail food animal consumption of medically important antimicrobials.

**Recent Findings:**

The features and relative success of the US efforts are examined alongside those of selected member states (Denmark and the Netherlands) of the European Union. Evaluation of efforts at all levels of US governance was complicated by shortcomings in prescribed data collection; nevertheless, available information suggests deficiencies in policy implementation and enforcement compromise the effectiveness of interventions pursued to date.

**Summary:**

The political will, robust systems for collecting and integrating data on antimicrobial consumption and use, and cross-sectoral collaboration that have been integral to the success of efforts in Denmark and The Netherlands have been notably absent in the USA, especially at the federal level.

**Supplementary Information:**

The online version contains supplementary material available at 10.1007/s40572-022-00351-x.

## Introduction

Most animal-source protein consumed in the USA comes from industrial food animal production (IFAP) operations [1]. These operations produce billions of cattle, pigs, chickens, and turkeys annually in the USA under typically large-scale, highly specialized, and densely stocked conditions that rely on an array of external inputs for their feed and maintenance [2]. In IFAP operations, antimicrobials have long been used to treat sick animals (“disease treatment”), and to control the spread of an identified disease among animals in close contact (“disease control”); they also are regularly given to groups of animals where none show signs of disease, but disease is anticipated (“disease prevention”) [3], [4]. Formerly, many antimicrobials also were given to US livestock (defined here as terrestrial animals produced for food) flocks and herds for production purposes (e.g., growth promotion), before the Food and Drug Administration (FDA) made those uses illegal at the start of 2017. The same products continue to be given legally via animal feeds to flocks or herds as disease prevention, however, often at dosages and for extended periods of time identical or nearly identical to the now-disallowed production uses [5, 6].

Most antimicrobials used in US animal agriculture (54%) are medically important antibiotics — i.e., antibacterial agents from the same drug classes relied upon for use in human medicine [7]. Numerous scientific bodies have expressed concern that widespread, non-human uses of these antimicrobials may contribute to significant declines in effectiveness of these or related medicines in human patients by selecting for resistant bacteria [8], [9]. The latter may occur when farmers, workers, and veterinarians are directly exposed to animals, or indirectly when people consume or handle raw poultry or meat products carrying drug-resistant bacteria, or have been exposed to farm-adjacent air, waters, and soils that can harbor reservoirs of transmissible antibiotic resistance genes and antibiotic-resistant bacteria themselves [10, 11]. Human studies provide evidence of elevated risks for colonization or infection with drug-resistant bacteria among those living near IFAP [2, 12–14].

In the USA, attempts to curtail agricultural antimicrobial use are not just a recent phenomenon; documented efforts date back to the 1950s [15]. Despite ongoing calls to limit avoidable agricultural usage, efforts to do so through regulatory means remain slowly or incompletely implemented, and subject to industry resistance [16]. In addition, sales of medically important antibiotics for use in food-producing animals have declined only modestly since 2009, when the US Food and Drug Administration (which oversees veterinary drug approvals) first began reporting them (Table 1). Sales actually rose by more than 8% from 2017–2020 [7].

**Table 1:** Antimicrobials sold for use in food-producing animals in the US, 2009–2020, in millions of kilograms of antibiotic active ingredient

	2009	2010	2011	2012	2013	2014	2015	2016	2017	2018	2019	2020	% change 2009*–*2020
Medically important	7.7	8.2	8.3	8.9	9.2	9.5	9.7	8.4	5.6	6.0	6.2	6.0	− 21.9%
Not medically important	4.9	5.1	5.3	5.7	5.6	5.9	5.9	5.7	5.4	5.5	5.3	4.3	− 12.6%
All	12.6	13.3	13.6	14.6	14.8	15.4	15.6	14.0	10.9	11.6	11.5	10.3	− 18.3%

Antimicrobial sales data are from the FDA Center for Veterinary Medicine, Summary Reports on Antimicrobials Sold or Distributed for Use in Food-Producing Animals, 2009–2020 (available: https://www.fda.gov/media/154820/download)

The purpose of this review is to describe US regulatory and other efforts to curtail antimicrobial use in IFAP at different levels of governance, contrasting them with efforts taken in select European Union (EU) member states, namely Denmark and The Netherlands. Successful approaches offer key lessons for the USA to draw upon, even if cultural, economic, and policy differences make their replication difficult, or even unlikely.

## The Threat of Antimicrobial Resistance and the Contribution of Animal Agriculture

The World Health Organization (WHO) describes antimicrobial resistance as one of the “biggest threats to global health, food security, and development today” [17]. Antimicrobial resistance, and especially bacterial resistance to antibiotics, is widely recognized as one of the most pressing health problems of the modern era. Recent estimates suggest nearly 1.3 M deaths worldwide are attributable to antimicrobial-resistant bacterial infections in 2019 [18, 19].

Antimicrobial use is the most important driver of increasing resistance [17, 20]. The global resistance burden is a collective function of different domains of antimicrobial usage across all countries. These domains include use in human medicine and companion animals, use in livestock production, and use on crops and orchard fruits [21–24]. Resistant bacteria are carried across international borders, but also travel among animals, humans, and their overlapping environments, necessitating a One Health approach [21, 25]. Genes that determine resistance can also be extensively shared among even unrelated bacterial species (e.g., extended-spectrum beta-lactamase (ESBL) genes). While these factors challenge attribution of resistance burden to a particular usage domain, a growing literature suggests that non-human sources of resistance are important to consider [26, 27].

Because antimicrobial use is the leading driver of resistance, reducing all unnecessary uses is a critical public health strategy. The available data, albeit somewhat limited, suggest that use in animal agriculture dwarfs that in human medicine in some high-income countries such as the USA [28]. Since the 1969 Swann Report, IFAP antimicrobial use has triggered consistent and ongoing public health concern. [29], [30].

Regulatory efforts in the USA are focused on addressing unnecessary usage of medically important antimicrobials in animal agriculture. The most recent data suggest around 66% of all medically important antimicrobials sold in the USA are intended for use in food animal production, primarily in swine and cattle (see Supplement). In addition to the large quantity of use in US production, some antimicrobial uses in IFAP involve administration in food or water to entire flocks or herds and for extended periods of time [31]. These factors add to the risk of selection for genes that confer drug resistance, as well as for the propagation and spread of potentially dangerous bacteria that carry this same resistance.

## Federal Regulatory Oversight of Agricultural Antimicrobial Use in the USA

US federal policies addressing use of medically important antimicrobials have been implemented voluntarily or incompletely. They also have contained significant loopholes, ensuring limited success in affecting overall use reductions.

In 2009, US sales of medically important antimicrobials labeled solely for therapeutic uses in food-producing animals accounted for 28% of all livestock antimicrobial sales, with the remainder (72%) being dually labeled for either therapeutic or “production” uses, e.g., growth promotion or feed efficiency [32]. Leading up to 2017, the FDA worked with the US pharmaceutical industry to voluntarily withdraw label claims for use of medically important antimicrobials in animal feeds on a flock- or herdwide basis for non-therapeutic or production (e.g., growth promotion, feed efficiency) purposes [3, 33]. The label claim withdrawal made uses for those purposes a violation of the Federal Food, Drug and Cosmetic Act [34]. Remaining feed uses of the same antimicrobials were placed under veterinary supervision [35]. This action to restrict growth promotion uses does not address their continued use under similar dosages and conditions for “disease prevention,” an indication that remains on product labels [36, 37].

The FDA explicitly defines antimicrobials used for disease prevention as therapeutic, even when that use occurs in flocks or herds without any sick animals or a specified etiologic agent. At least thirteen medically important antimicrobials are FDA-approved for use in feed for disease prevention with no clear time (“duration”) limits — meaning groups of animals are potentially exposed to them on a near-continuous basis [31, 38]. In contrast, the European Parliament voted in 2019 to forbid antimicrobial use for disease prevention, effective January 28, 2022; the World Health Organization also has determined that the use of medically important antimicrobials for disease prevention is unnecessary (except under very exceptional circumstances), and thereby detrimental to public health [39].

The FDA’s current approach relies on individual veterinarians to decide, on a case-by-case basis, whether ongoing antimicrobial use, including for disease prevention, is appropriate or “judicious.” The American Veterinary Medical Association (AVMA) also states that the prescribing veterinarian determines if and when antimicrobials have been used judiciously in animals (American Veterinary Medical Association, 2021). The FDA does not systematically report information on veterinary antimicrobial prescriptions or directives at a national level; thus, any responsibility for oversight of veterinarian prescribing patterns would fall to the autonomous state veterinary medical boards. Furthermore, we can find no examples that records of veterinary prescriptions or directives are being collected, maintained, or analyzed at the state level, apart from Maryland and California, as is later discussed. Even in these states, collection and analysis activities are limited.

## Surveillance of Antimicrobial Use in US Food Animal Production

Despite antimicrobial use being the chief driver of resistance, US federal agencies do not collect or report farm-level use data nationally, despite recommendations for such collection from the Government Accountability Office [40–42]. Individual producers rarely report data on their own use of antimicrobials, which they typically consider confidential business information. In the absence of these data, regulators collect and report antimicrobial sales data as a proxy for antimicrobial usage. Since 2010, the FDA has published annual reports summarizing the previous year’s antimicrobial sales for use in food-producing animals, most recently for 2020 [7]. The European Surveillance of Veterinary Antimicrobial Consumption (ESVAC) program also has relied on sales data sine 2009 to track antibiotic consumption by food-producing animals in up to 31 different countries [43]. In contrast to the lack of available use data for terrestrial food animals, two US states (Maine and Washington) collect these data in the process of permitting salmon aquaculture operations [44].

Initially, the FDA’s annual summary sales reports were limited in the data and analysis they presented. Since 2016, based on changes in reporting requirements, the reports include sales by animal species, route of administration, usage indication, dispensing status, and combined route of administration and drug class [35]. One notable limitation to those data is that the same antimicrobial-containing products may be marketed for multiple indications, and/or for use in multiple animal species; estimates of sales intended for use in a single species are inexact as a result. Sales data also are reported only at the national level, precluding analyses informing spatio-temporal trends or facilitating cross-producer comparisons. Ideally, antimicrobial consumption data would be made available at a higher level of temporal (e.g., month or quarter) or spatial (e.g., by producer, individual state, or USDA region) resolution in order to enhance comparisons with surveillance, e.g., National Antimicrobial Resistance Monitoring System (NARMS), and clinical data on resistant organisms.

Before 2017, feed mills sold pre-mixed feeds containing antimicrobials over the counter, even over the internet, without veterinary oversight or a prescription. Under FDA Guidance #213 and its Veterinary Feed Directive (VFD) final rule, however, medically important antibiotics administered in feed or drinking water were brought under veterinary oversight by requiring either a VFD or prescription. Since the VFD final rule was enacted, feed mills have been required to receive a veterinary order, or VFD, before delivering feeds containing medically important antibiotics to producers [45]. The VFD rule applies to antimicrobials mixed into animal feed and requires feed mills, along with prescribing veterinarians and their clients, to keep certain records for 2 years, including identifying information about the veterinarian, client, and patient[s], premises, drug name, indication, dosage, and duration [46]. At the same time, neither veterinarians nor producers are obligated to maintain records of non-feed antimicrobials they may have prescribed or administered — those given topically, for example, orally, or by injection to individual animals, or administered via drinking water supplies to entire flocks or herds. Since enactment of the VFD Rule, the FDA has rarely exercised its authority to collect and inspect retained VFD records, doing so only once from 2016 to 2018 for 278 feed distributors, 21 animal producers, 14 veterinarians, and 5 entities serving two or more of those roles [47]. No antimicrobial use data from that effort were made public however, not even data that had been aggregated or otherwise rendered anonymous.

## Trends in Veterinary Antimicrobial Consumption in the USA

**Fig. 1:**
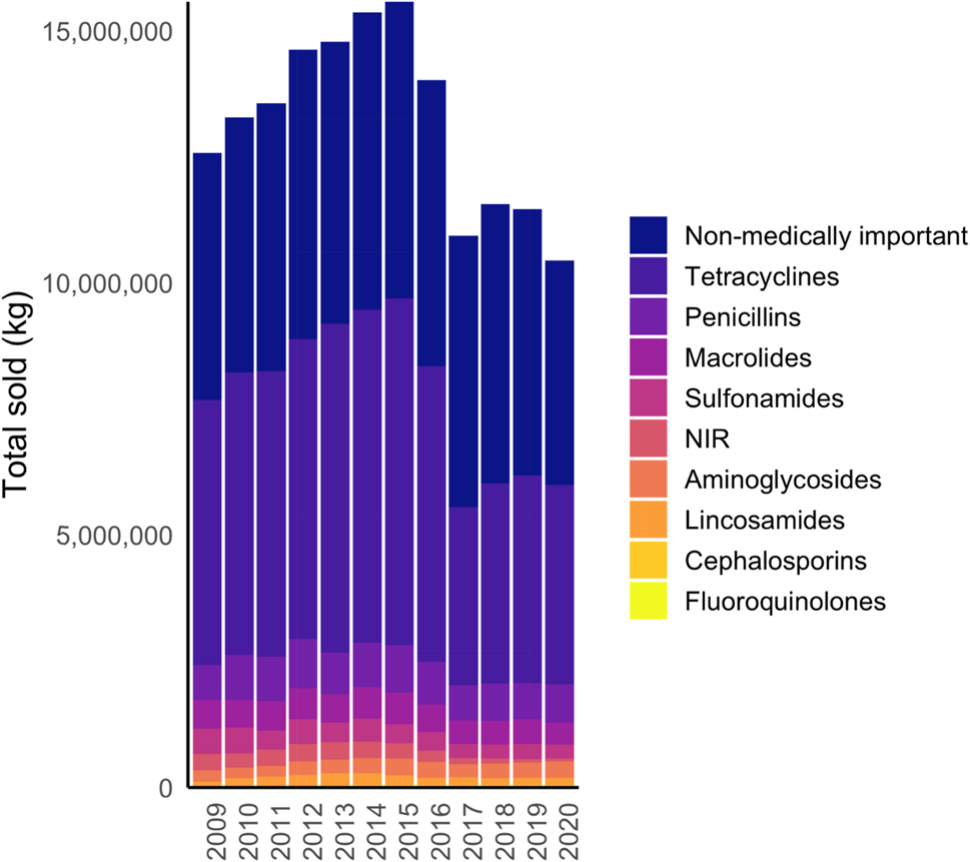
Total US livestock antibiotic sales in kilograms, by class and year. NIR, Antimicrobial drug classes with fewer than three distinct sponsors of approved and actively marketed animal drug products are reported collectively as “Not Independently Reported” (NIR)

Nearly 73% of all antimicrobials sold for use in US animal agriculture in 2009 carried at least some production uses (e.g., growth promotion, feed efficiency) on their labels [32]. Based on a decade’s worth of FDA summary reports on antimicrobial sales, the consumption of antimicrobials by food animal production has declined by about 18% overall since 2009 (Fig. 1 and Table 1), with sales of non-medically important classes falling 13% and sales of medically important classes declining 22% [7]. While sales of sulfonamides and tetracyclines have had the most marked declines (reduced by 44% and 25%, respectively, from 2009), sales of some drug classes critically important to human medicine have risen, including cephalosporins (up 30% from 2009) and fluoroquinolones (up 60% from 2013).

These longer-term trends obscure some important shifts over shorter time frames. Overall sales kept rising from 2009 to a peak in 2015, for example, despite the AVMA’s and FDA’s ongoing promotion of “judicious use.” Modest declines occurred in 2016 and 2017, as the FDA extended veterinary oversight to medically important antimicrobials in feed, and also worked with pharmaceutical companies to voluntarily stop marketing these same products for growth promotion or feed efficiency; at the outset of 2017, the latter uses were no longer legal due to the withdrawal of the label claims by the drug companies.

The FDA only began reporting sales of medically important antimicrobials by the animal species for which they were intended in 2016, when directed to do so by Congress [48]. From 2016 to 2020, sales of medically important antimicrobials in particular fell (kilogram of active ingredient sold) for all four major species, but they fell further for chicken (− 72%) than for turkey (− 9%), swine (− 22%), or cattle (− 32%) production. Potential explanations for why declines for chicken outpaced other sectors include the following: the shorter lifespan (35–42 days) of broilers, translating to fewer days with opportunities for antimicrobial use; the industry’s higher use of non-medically important alternatives such as ionophores, vaccines, and probiotics, shifting market demand due to changing consumer preferences [49] and the increasing popularity of No Antibiotics Ever production systems [50], the marginal cost-effectiveness of routine antimicrobial use in chicken flocks [51], and the chicken industry’s anticipation of tightening restrictions from the FDA [52].

Sales of medically important antimicrobials for all food animal production rose 11% between 2017 and 2019, driven by a 28% rise in sales for swine production (levels declined 6% again from 2019 to 2020). This rise may stem partly from several recent outbreaks in US swine production, including porcine reproductive and respiratory syndrome (PRRSV) and porcine epidemic diarrhea (PED), given the potential for antibiotics to be used before viral infection is confirmed or in cases of secondary bacterial infection. Meanwhile, the sales of non-medically important antimicrobials fell 17% between 2017 and 2020 [37].

Any changes in antimicrobial consumption (sales) over time, whether in livestock production as a whole, or in a particular livestock sector, are more comparable if normalized or biomass-adjusted (e.g., adjusted by the estimated weight of the animal population most likely exposed to those drugs). The European Medicines Agency (EMA), for example, standardized and began to extensively use a biomass-adjustment metric in 2010, expressed as mg/kg or mg per population correction unit (PCU) [53]. By contrast, the FDA did not issue its own draft method for doing biomass-based adjusting annual sales information until 2017 [4]; final implementation of that method has been further delayed, since the final method has yet to be published [54].

The trends in US antimicrobial consumption suggested by the sales data in Table 1 can be confirmed by applying the EMA’s biomass adjustment methodology; the attached Supplement details the use of publicly available data to calculate PCUs, as well as the mg/kg rate (or intensity) of consumption of medically important antibiotics in US food animal production in 2020. The approach was the same for 2016 through 2019, though calculations cannot be made for years prior to 2016, since the FDA had not yet begun publishing sales by individual animal sectors.

Table 2 details changes in the intensity of medically important antibiotic use in US food animal production since 2016. It shows that biomass-adjusted consumption of these drugs in US livestock production overall fell significantly from 2016 to 2017, before rebounding 4.5% over the next 3 years, to a consumption intensity of 170.8 mg of antimicrobial/kg of livestock in 2020. This 3-year increase has been largely fueled by higher rates of consumption of these drugs in US swine and cattle production, which rose 12.1% (to 267.9 mg/kg) and 5.3% (to 161.3 mg/kg), respectively.

**Table 2:** Consumption of medically important antimicrobials by livestock sector from 2016–2020, in milligrams of antimicrobial active ingredient consumed per kilogram of livestock

	2016	2017	2018	2019	2020	% change 2016–2020	% change, 2017–2020
Chicken	55.5	29.6	24.2	20.7	15.2	− 72.7	− 48.8
Cattle	232.6	153.1	162.8	163.1	161.3	− 30.7	5.3
Swine	380.2	239.0	272.9	285.1	267.9	− 29.5	12.1
Turkey	478.5	427.0	435.9	435.8	476.6	− 0.4	11.6
Overall	249.8	162.0	173.0	174.9	170.8	− 31.6	5.5

Antimicrobial sales data are from the FDA Center for Veterinary Medicine, Summary Reports on Antimicrobials Sold or Distributed for Use in Food-Producing Animals, 2009–2020 (available: https://www.fda.gov/industry/animal-drug-user-fee-act-adufa/adufa-reports). Methods supporting the calculations are provided in the Supplement.

## US State- and Local-Level Interventions

In the absence of US federal actions to track and reduce farm-level use of medically important antimicrobials, some states and localities have implemented more aggressive policies. Two states with large livestock industries — California (dairy, beef, and poultry) and Maryland (poultry) — have passed laws restricting the regular use of medically important drugs in meat and poultry production. Both state laws also require the collection of livestock antimicrobial use data. Beyond approaches at the state level, the city of San Francisco has also recently implemented a market-based approach to understanding patterns of antimicrobial use.

### *California*

With 39.5 million residents, California is the country’s most populous state and ranks among the nation’s largest producers of dairy cows and cattle and broiler and layer chickens [55], [56]. California was the first state to pass legislation to address agricultural antimicrobial use, in 2015 [57].

An earlier bill (SB835) that merely replicated federal guidance to end some uses of medically important drugs in groups of animals for growth promotion—but continued to endorse other, virtually identical uses for disease prevention in the absence of any sick animals—failed [58]. Despite early support for this initial bill from agricultural industry trade associations and veterinarian groups, and some health groups, it had faced stiff opposition from various public health, consumer, environmental, and progressive farming organizations. In response to the opposition, Governor Jerry Brown eventually vetoed the bill [59]. Senate Bill 27 (SB27), which revised the previous bill to significantly curtail the use of medically important antimicrobials for disease prevention, was introduced, passed, and then signed into law by Governor Brown later in 2015.

SB27 prohibits use of medically important antimicrobials in livestock “in a regular pattern” unless two conditions are met: (1) use is ordered in a prescription or feed directive from a licensed veterinarian and (2) use is necessary to treat a disease or infection, to control the spread of disease or infection, or in relation to surgery or a medical procedure [60]. Limited preventive use of medically important antimicrobials is also allowed, but only when necessary to address an “elevated risk” and never, without exception, in a “regular pattern” [61]. The law, which went into effect on January 1, 2018, also mandates California’s Department of Food and Agriculture (CDFA) to gather antimicrobial sales and on-farm usage data, as well as samples from operations across the state and food chain.

Stakeholders have suggested that implementation of the law has not met its intended goals. Because the actual law prohibits the use of medically important drugs in a regular pattern, while the CDFA’s initial communications suggested the opposite, the Natural Resources Defense Council (NRDC) has asserted that the agency’s misinterpretations of the law created the potential for confusion among California’s producers and also heightened the possibility that producers would continue to illegally use these drugs in a regular pattern. CDFA attempted several iterations of its guidance documents before a static version of the guidance was issued in summer 2019 [57, 62, 63], and communication of these revisions to industry and producers may also have lagged.

Data collection and reporting in California also lagged mandated milestones under SB27. Although the 2015 law required a system for comprehensive data collection to be in place by the time the law went into effect in January 2018, that did not happen. As a result, CDFA’s first report on these anticipated data was not released until late 2019, and was incomplete [64]. Also at odds with the 2015 law is the fact that CDFA’s public reporting only reflected the amount of medicated feed sold in the state, rather than the specific quantities of antimicrobials mixed into the feed, or the number of animals for which the feed was intended. CDFA also has emphasized voluntary surveys to collect data, which are prone to selection bias and information bias and thus may challenge the generalizability and reliability of results. Beyond the reporting limitations, the law also treats much of the information provided to CDFA as confidential, preventing external analyses [61]. Regardless, baseline data were never collected; those missing data are essential to evaluate whether antimicrobial usage reductions occurred after the law was enacted.

Passage of California’s law in 2015 signified an important forward step towards restricting the unnecessary use of medically important antimicrobials in the state’s food producing animals. However, gaps in the law, delayed and uneven implementation, and lack of open data have collectively undercut the law’s intent, and may have also impacted the law’s effectiveness in tracking and helping to identify potentially disallowed uses of medically important antibiotics in food-producing animals.

### Maryland

Maryland has slightly more than 6 million residents, but is among the most densely populated states (241 residents per km^2^) [56]. Most of the state’s food animal production is concentrated on the DelMarVa peninsula, shared between Delaware, Maryland, and Virginia, and on which Maryland alone produces 289 million broilers annually [65, 66]. With 5000 chicken houses capable of producing 149 million broilers at any time, broiler production density on the DelMarVa peninsula (466 birds/km^2^) is one of the highest in the USA, which itself is one of the world’s largest exporters of poultry products [67].

Maryland followed California with a similar law (SB422) in 2017 that bans most “regular pattern” use of medically important antimicrobials, although without California’s reporting provisions. The Maryland Department of Agriculture’s (MDA’s) initial proposed regulations, however, were criticized by stakeholders for defining prohibited “regular pattern” use to mean only off-label pulse dosing — essentially only prohibiting uses that were already illegal [68]. In 2019, the legislature added more specific directives and new, detailed reporting requirements to the existing law [69].

At the time of publication, Maryland’s SB422 law is now stronger in several key respects than California’s SB27 and the California Department of Food and Agriculture’s updated guidance from 2020. First, it includes language limiting the MDA’s authority to soften the intent of the law during implementation. The law also further clarifies the scope of prohibited uses of medically important antimicrobials in a “regular pattern”: namely, these drugs cannot be used repeatedly in the same animal or group of animals or as a standard operating procedure—for example, as a management tool or strategy (e.g., routinely at a certain date, season, or age, such as at birth or weaning), or when animals have changed locations. The law clarifies that the elevated risk of disease that could justify the exception of preventive use must be unusual; it “does not include a risk typically or frequently present under normal or standard operating conditions” [69].

Additionally, Maryland’s new reporting requirements are considerably more detailed than California’s. They require MDA to annually report on the following: (1) the total number of animals raised on farm operations covered by the provisions governing medically important antimicrobial drugs, categorized by species and production class; (2) the specific antimicrobial active ingredients and classes of antimicrobial active ingredients used; (3) the total weight of antimicrobial active ingredients used; (4) indications for which veterinarians prescribed medically important antimicrobial drugs; and (5) patterns of use for medically important antimicrobial drugs, including duration and seasonal variation. SB422 is now the strongest state law concerning agricultural antimicrobial use in the US [70]. In 2022, MDA released the second annual report under the strengthened law to the state legislature, the contents of which, largely conformed with that law's requirements [71].

### San Francisco, CA

In 2017, San Francisco became the first US city to take municipal action related to public health concerns around widespread antimicrobial use in food animal production. It passed an ordinance requiring large grocery chains to report two types of antimicrobial information for meat products sold in their stores: first, information about the antimicrobial use policies of the producers supplying these products, regarding why and under what circumstances they might have used antimicrobials in that production; and second, actual numbers, i.e., the number of animals raised by those same producers, and the quantities of antimicrobials they used in that production [72]. Under the ordinance, grocers and meat producers are jointly responsible for the required data. They can receive a time-limited waiver if able to present evidence showing that data, for specific products and information, are not feasible to report without significant hardship. Guidance and regulations for implementing the ordinance make it clear that a waiver request must include a plan for future acquisition and submission of that information [73].

Enormous consolidation within the US supermarket sector means that retailers operating in large cities are largely the same nationwide, albeit with some minor regional variation. San Francisco’s approach therefore has the potential to improve consumer reporting around antimicrobial use policies and practices of meat companies producing at a national, even global, scale.

At the time of publication, San Francisco’s Department of the Environment had reported 2 years’ worth of collected data [74, 75]; these reports illustrate the ordinance’s potential, and highlight challenges in obtaining antimicrobial use data from food animal producers individually, and as an industry, as well. At a relatively high response level, grocery chains have answered questions about producer policies around antimicrobial use — e.g., indications for which drugs are allowed, with policy responses improved by the second year. Even then, however, the rate of reporting of figures on antimicrobial use has remained poor; for 2019, for example, grocers reported antimicrobial use data for only one of 29 beef products supplied to them by the four companies now controlling around 80% of beef processing, nationally [74, 75]. Similarly, they reported use for only one out of 18 pork producers. In contrast, complete antimicrobial use data were reported for 40% and 74% of all chicken and turkey products being sold by these grocers, respectively.

The San Francisco reports suggest that big grocery chains could work with their suppliers to ensure more complete reporting of information around antimicrobial use, starting with the companies supplying the store-branded products over which they have full control of the product specifications, e.g., suppliers of Target’s Good & Gather™ or Archer Farms™ chicken. For example, staff at SF Environment report that the five major grocers within the city – Albertson’s, Costco, Krogers, Target, and Trader Joe’s—collectively sell 133 store-branded meat products derived from animals given antimicrobials. Only two of the five, Kroger and Target, have provided *any* data on drug use for their own, store-branded products, and each of the two companies supplied data for only a single type of (unspecified) product [74].

### US Case Study Conclusions

The US chicken industry has made rapid strides to eliminate the routine use of medically important antimicrobials, and that progress is now being described in both government and industry reports. A poultry trade industry publication recently announced 60% of US broiler chickens are now raised without any antimicrobials [50], [37]. Meanwhile, the FDA’s annual sales reports suggest that sales of medically important antimicrobials for US chicken production (as a portion of all such drugs consumed by food-producing animals) dropped from 6.1% in 2016 (FDA’s first year of reporting sales by species) to just 2.4% in 2020; over that same stretch of time, 2016 to 2020, the estimated share of all food animal antimicrobials sold for use specifically in swine production rose from 37.5 to 40.8% [7]. Antimicrobial consumption on a biomass-adjusted basis also differs between chicken and other food animal sectors. As shown in Table 2, the consumption rate for US production of broiler chickens had fallen by 2020 to just under 15 mg/kg of chicken. The comparable weight-adjusted consumption rates for other US food animal sectors range from 161 mg/kg in cattle production to 268 mg/kg in swine production and 477 mg/kg in turkey production (see Supplement). Therefore, while overall and medically important antimicrobial use has declined in chicken, use in other animal sectors remains high.

Major challenges persist in the implementation of various laws and policies in the USA pertaining to antimicrobial use in food animal production. Implementation of California’s law has fallen especially short of its intended goals. The full implementation of San Francisco’s municipal ordinance, despite a strong effort, also has been hampered, especially given the beef and pork sectors’ unwillingness to provide antimicrobial use information. Maryland’s newly strengthened law is still in the early stages of implementation but provides the best US example of a governmental entity acting as its lawmakers intended. Its requirements for antimicrobial use reporting, largely based on state-collected veterinary feed directives, suggest that if this information were collected and reported at a national level, it could shed substantial new light around farm-level antimicrobial use — transparency that is not afforded by the FDA’s annual reports on antimicrobial sales alone. These examples carry policy significance for other states (including Oregon, Illinois, and New York, where similar bills have been introduced) aiming to address antimicrobial consumption in food animal production.

## European Union Approach to Antimicrobial Use Reduction

The 1998 *Copenhagen Recommendations* called on the European Union and member states to recognize antimicrobial resistance as a major problem [76]. This call was followed by the first European Community Strategy Against Antimicrobial Resistance, which aimed in part to phase out non-medicinal uses of antimicrobials in both human and food animal settings [77]. As of 1999, antimicrobials important to human medicine were no longer allowed as additives to animal feed for the economic purpose of growth promotion; effective January 1, 2006, growth promotion uses of the remaining (non-medically important) antimicrobials were also banned [78, 79]. From 2007 to 2013, the EU embraced and implemented an animal health strategy and action plan that "Prevention is Better than Cure"; a central goal was to employ disease surveillance and investments promoting animal health in order to prevent unnecessary infections, thereby avoiding unnecessary antimicrobial use [80].

In 2011, the EU issued a new action plan, again reinforcing the need for a region-wide approach to antimicrobial resistance, based in One Health [81]. A third, updated EU action plan, adopted in 2017, again reflects Europe’s commitment to a broader set of actions, “tackling AMR more comprehensively on the basis of improved data collection, monitoring and surveillance,” and extending the One Health approach to include the environment [82]. New EU-wide legislation on veterinary medicines also was adopted in 2018, effective January 28, 2022 [83]*.* That legislation includes important elements to increase transparency, such as the mandatory monitoring of antimicrobial use on farms. It also calls for more prudent and responsible antimicrobial use. A third element is a new ban on antimicrobial use for disease prevention, and a fourth is the establishment of a process whereby, once the EMA finalizes a list of drugs it recommends not be used at all in food animal production, certain of those antibiotics could be withdrawn from use.

**Fig. 2:**
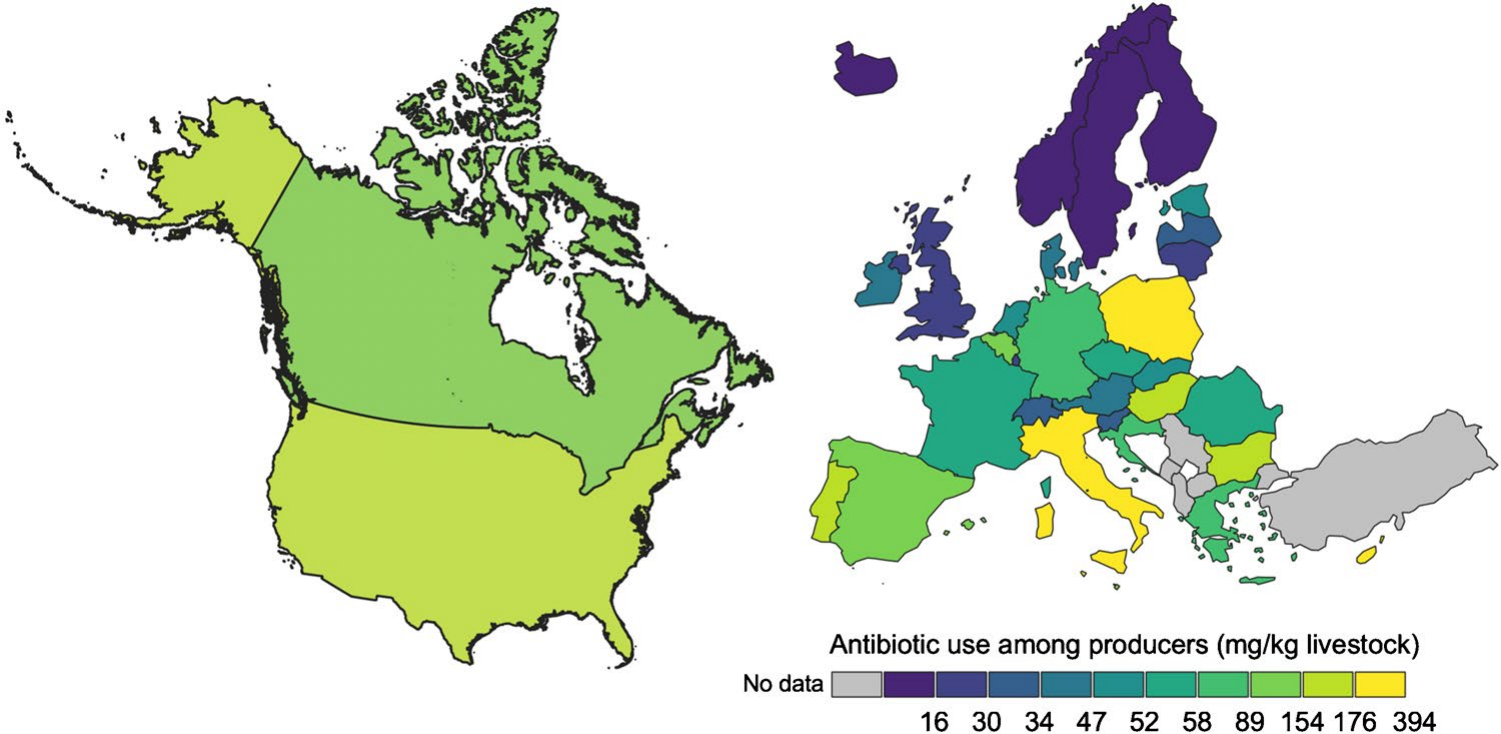
Map of antibiotic use among producers in North America and Europe in mg antibiotic per PCU of livestock. Total consumption of medically important antimicrobials for terrestrial food-producing animals (cattle, dairy, swine, and poultry). Mg/PCU data and methodology for all European countries are from [43] “Sales of veterinary antimicrobial agents in 31 European countries in 2020.” (EMA/24309/2020), Available: https://www.ema.europa.eu/en/documents/report/sales-veterinary-antimicrobial-agents-31-european-countries-2019-2020-trends-2010-2020-eleventh_en.pdf. Canadian mg/PCU data are from 2018, and available from Public Health Canada, Canadian Antimicrobial Resistance Surveillance System, 2020 Update. Available: https://www.canada.ca/content/dam/hc-sc/documents/services/drugs-health-products/canadian-antimicrobial-resistance-surveillance-system-2020-report/CARSS-2020-report-2020-eng.pdf; US antibiotic sales data (in kg/year) are from the 2020 Summary Report on Antimicrobials Sold or Distributed for Use in Food-Producing Animals. Available: https://www.fda.gov/media/133411/download. A description of the data sources and method for converting these US sales data to mg/PCU is provided in the Supplement

**Fig. 3:**
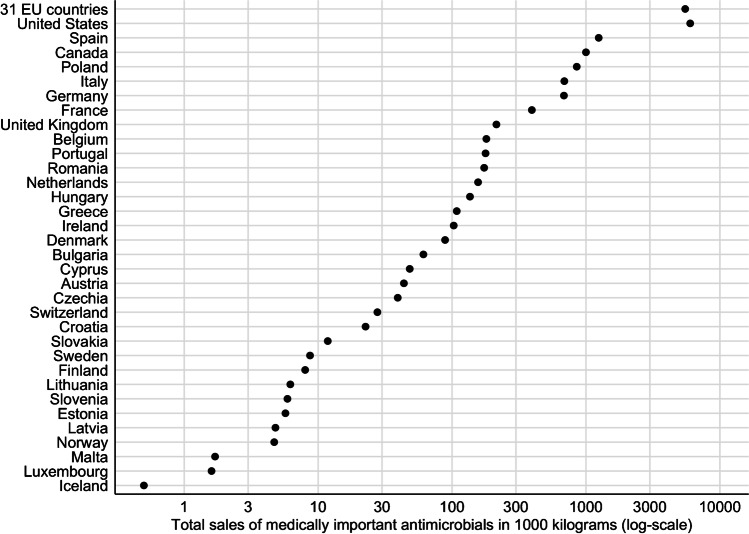
Total sales of medically important antimicrobials in 1000 kg in North American and European countries. Total sales of medically important antimicrobials for terrestrial food-producing animals (cattle, dairy, swine, and poultry) in 2020, except for Canada (whose data are from 2018)

Antimicrobial sales for food animal production across the entire EU have dropped steadily. Consumption by these sectors fell 43.2% on a biomass-adjusted (mg/PCU) basis from 2011 to 2020, across the 25 countries collectively that had reported data each year to the EMA [53]. As of 2020, the rate of antibiotic consumption in food animal production across all of Europe is now reported as 91.6 mg/PCU [53]. Although uses among species varies significantly from country to country, it nevertheless seems notable that medically important antibiotics are consumed in the USA at a rate (170.8 mg/PCU) roughly 86% higher than their consumption by counterpart industries across 31 European countries, collectively (Figs. 2 and 3). Even within the EU, the much higher than average declines in antimicrobial consumption in some individual countries suggests that additional reductions in antimicrobial use are possible in the region [43]. Bounded by European regulations, EU member countries have used different approaches and timelines to bring about such reductions in antimicrobial use. National rules also vary significantly in how comprehensively they aim to monitor and curtail that use.

A two-part report from the European Commission’s Directorate General for Health and Food Safety summarized results from fact-finding missions carried out in member countries, beginning in 2016. Drawing upon certain successful measures taken, including in Denmark and The Netherlands, the reports identify a set of common policies which, if implemented at all levels, can result in antimicrobial use reductions of more than 50% [84, 85].

Historically, human antimicrobial use in both Denmark and The Netherlands has been lower than the EU average. Both countries have prominent, export-oriented food and agriculture industries involving intensive, industrialized livestock production systems [86]. They also are recognized as being among the European countries acting early, comprehensively, and successfully to reduce antimicrobial consumption and use by their important food animal sectors.

Common to each country’s approach were the following key elements. Strict new rules were established and enforced, as were antimicrobial use reduction targets. New research and surveillance programs were enacted and were critical for reaching these targets and for tracking the subsequent changes in the prevalence of antimicrobial resistance. Thirdly, additional steps were taken, including those to (1) improve tracking of antimicrobial consumption, as well as resistance in bacterial samples collected from human and animal populations and from food products; (2) integrate results from surveillance of antimicrobial sales/consumption and resistance into a single annual report; and (3) use those tracking systems to help set benchmarks for veterinarians and/or farm-level antimicrobial use, and to inform subsequent policy interventions over time [86].

### Denmark

Denmark has around 5.8 million human inhabitants. Around 5000 Danish pig farms produce approximately 28 million pigs per year, more than any US state except Iowa. Most of the production (90%) is exported, making Denmark one of the world’s largest pork exporters. Dairy, poultry, and egg production are other important agricultural sectors in Denmrak, with around 60% of poultry meat being produced for export (Danish Agriculture and Food Council, 2020).

In 1995, the Danish government initially acted to phase out *all* antimicrobial use in herds for disease prevention [87]; 1 week later, a second order was enacted banning the veterinary use of avoparcin — a glycopeptide drug sold as a swine and poultry feed additive for growth promotion, the only approved use — as a potential human threat [88]. The second action was triggered by new evidence linking avoparcin’s use in food animals to the emergence of a major animal reservoir of Gram-positive enterococcal bacteria (VRE) carrying high-level resistance to another glycopeptide, vancomycin. Vancomycin was used in Danish hospitals, as elsewhere, as a drug of last resort for treating serious infections caused by gram-positive bacteria. However, it was sparingly used on human patients, and not at all in food animals [88].

Reservoirs of VRE in animals in Denmark seemed to signify that cross-resistance was occurring. That is, avoparcin use in animals was cross-selecting for vancomycin resistance, since both were glycopeptides. The evidence was sufficiently compelling that just 2 months later, avoparcin’s use for growth promotion was banned across the entire EU, effective in 1997 [89–91]. The two early bans drew broader attention to what had been very widespread antimicrobial use in food animals for non-medicinal reasons, such as growth promotion, and the ensuing risks to the human population [92]. Prior to these actions, for example, the antimicrobials sold and used for growth promotion accounted for more than half of all such drugs consumed in food animal production in Denmark [92].

Also in 1995, Denmark established the Danish Integrated Antimicrobial Resistance Monitoring and Research Program (DANMAP), the first national platform for systematic and continuous monitoring of antimicrobial use and resistance in animals, food products, and humans [92, 93]. DANMAP-generated data showed that Denmark’s total use of antimicrobial growth promoters continued to increase after 1995, despite the avoparcin and disease prevention bans, until the spring of 1998. Only then, after significant media attention, public concern, and the ensuing political pressure, did Danish pig and poultry producers initiate a voluntary end to their use of all antimicrobial growth promoters in finisher pigs and broiler chickens [89, 92].

A WHO-convened expert panel in 2003 reviewed Denmark’s experience ending antimicrobial growth promotion in poultry and swine. It concluded antimicrobial consumption in food animal production overall subsequently dropped by more than 50%; levels of drug-resistant bacteria in animals were “dramatically reduced”; and, there was no increased cost for poultry producers, and negligibly higher costs (~ 1%) for swine producers, offset by the likely benefits to public health and consumer trust from ending this unnecessary use of antimicrobials [94].

Aarestrup [89] has attributed Denmark’s success to three key elements: the political will to enforce regulations, cross-sector collaboration between farmers, researchers, and authorities; and the availability of data showing that antimicrobial consumption in food animals was becoming a problem. DANMAP has been instrumental in producing the latter data, but also for showing the full benefit of actions taken to curtail antimicrobial consumption in food animal production, including changes in the prevalence of key types of antimicrobial resistance in food animals and in the human population.

After the *Copenhagen Recommendations* were issued, Denmark established the VETSTAT system in 2000 (Stege et al. 2003). VETSTAT collects monthly data on all antimicrobials used in food animal production, data originating from veterinarians, veterinary pharmacies, and feed mills. This capability has been critical for assessing antimicrobial consumption at the farm level, including where (farm, region), when (time of year, animal age), and how (indication, administration method) antimicrobial use occurs. VETSTAT built upon another early strategy Danish experts consider important that “decoupled” veterinary prescribing and antimicrobial sales and distribution. Veterinarians once were paid to prescribe and sell antimicrobials. Only veterinary pharmacies can dispense the drugs today, removing a possible incentive for overprescribing.

Robust data collection on antimicrobial prescribing, distribution, and use, at a granular level, is what enabled Danish authorities to later implement their “yellow card” system. This program identifies pig producers whose antimicrobial use is higher than government-established thresholds. By setting iterative new thresholds, and targeting producers who exceed them, Denmark has further reduced overall drug use by its livestock industries [86].

### The Netherlands

Denmark and the Netherlands have a similar land area, but the Netherlands is approximately three times more populated, with 17.4 million inhabitants (517 residents per km^2^). The Netherlands raises twelve million pigs at any one time, along with 100 million broilers and laying hens, four million cows and veal calves, and 1.5 million sheep and goats (Netherlands Statistics, 2020). Intensive pig and poultry farms are remarkably concentrated in parts of the country, oftentimes near urbanized areas, which has contributed to public health concerns about possible transmission of antimicrobial resistance from farms to humans [14, 95]

Even after the European Union’s 2006 ban on all antimicrobial use for growth promotion, high levels of use persisted in food animal production in the Netherlands [96]. Two events helped change that situation. First, there was the 2005 discovery that livestock-associated methicillin-resistant *Staphylococcus aureus* (LA-MRSA) was highly prevalent in Dutch pigs, with transmission to farmers, their family members, and veterinarians [97]. This discovery led to formation of a Taskforce on Antibiotic Resistance in Animal Husbandry, and development of national action plans. Despite this, no new regulations or strict reductions targets around antimicrobial use were initially formulated.

A second event occurred a few years later: the nation’s retail poultry supply was discovered to be widely contaminated with extended-spectrum beta-lactamase (ESBL) producing bacteria. A prominent news story, alleging a direct link to the death of an infected person, heightened public health concern around antimicrobial use in animals [98]. In quick response, and with 2009 as its baseline, the Dutch government soon set targets to reduce antimicrobial consumption in food animals 50% by 2013, and 70% by 2015 [96]. Key policy changes soon followed, including prohibition of antimicrobial use for disease prevention,veterinary guidelines for responsible antimicrobial use also were generated. The independent Netherlands Veterinary Medicines Authority, or SDa, was established in 2010 to collect reliable antimicrobial usage and prescription data from individual farms and veterinarians, and to issue annual reports on it. SDa was charged with setting benchmark indicators for antimicrobial consumption, based on analysis of these data [99]. After setting such benchmarks for farm-level use, the SDa took aim to ensure additional transparency around patterns of antimicrobial use by setting benchmark indicators for veterinary prescribing as well [96, 100].

With the new policies, antimicrobial consumption (sales) in food animals during 2019 had decreased by 69.6% relative to 2009, indicating that the initial 70% reduction target had effectively been met. As in Denmark, declines in antimicrobial use have been somewhat matched by lower levels of bacterial resistance in animals. The MARAN (Monitoring Antimicrobial Resistance and Antibiotic Use in Animals in the Netherlands) surveillance system, for example, shows that prevalence of resistance among commensal *Escherichia coli* has fallen [101, 102].

Some top-tier antimicrobials (among them fluoroquinolones, and 3rd/4th generation cephalosporins) are hardly used anymore; since 2017, however, veterinary use of colistin has risen 62% (Netherlands Veterinary Medicines [103]. When the WHO decided in 2018 to move polymyxins, including colistin, into the category of “Highest Priority Critically Important Antimicrobials,” the SDa set a new target to eliminate colistin use in food animals.

### EU Case Study Conclusions

As previously noted, a recent European Commission report concludes that by adopting a suite of new regulations and policies already implemented successfully elsewhere, EU member countries with continued high-level antimicrobial consumption in food animals can likely achieve overall reductions of 50% or more [84, 85]. The same report identified no additional cost to producing chickens without the use of antimicrobials for disease prevention, growth promotion, or other production purposes; in swine, any additional costs were found to be either negligible, or more than outweighed by the benefits of avoiding these antimicrobial uses to food safety and public health (in swine).

The Netherlands’ successful approach suggests that simply banning antimicrobial growth promotors or signing multi-stakeholder action plans without compulsory targets is likely to be less effective or ineffective in reducing overall antimicrobial usage. Compared to Denmark, authorities in the Netherlands adopted more of a facilitation role while placing primary responsibility for reducing veterinary antimicrobial use on private parties, through self-regulation [96]. Notably, the country met its 70% use reduction target despite not decoupling veterinary prescribing and dispensing, as Denmark did [86].

## Key Differences in Approach and Effect Between the EU and the US

The interventions adopted to reduce antimicrobial use in food producing animals differ substantially between Denmark, the Netherlands, and the USA. Despite calls from around the globe to address antimicrobial use in this sector, European regulators took action much earlier than their US counterparts to restrict antimicrobial use for growth promotion and to enact bans on specific drugs, although approaches initially varied by country. For example, Denmark ended all such uses in 1995; in January 2022, the same prohibition went into effect across the entire EU. Meanwhile, the routine use of these medicines for herdwide disease prevention continues in the USA, even in the absence of any diagnosed disease, and the FDA considers it to be part of “therapeutic” usage.

Additional restrictive measures taken within Europe, sometimes by individual member states, have been discussed but never implemented in the USA. These include, for example, adoption of certain drug-specific usage targets; preservation of specific antibiotic classes for human-only use; and measures to ensure less frequent prescribing of WHO AWaRe “Watch Group” or “Reserve Group” antimicrobials [104].

Another significant difference has been the establishment in certain EU member states of comprehensive antimicrobial use surveillance systems with regular reporting, such as DANMAP, VETSTAT, and MARAN. These tracking systems have facilitated better drug stewardship, oversight of veterinary prescribing, and measurement of impact across several indices (i.e., live animals, retail meat, and human infections). In Denmark and Netherlands, meaningful veterinary oversight has been a key factor in limiting untargeted preventive uses of antimicrobials. The data generated by surveillance systems tracking on-farm antimicrobial use as well as resistance has been a critical component of their success, making possible Denmark’s yellow-card program, for example.

Calls for the creation of a similar system in the USA have gone largely unrealized. Notably, the lack of national data on antimicrobial usage at the production level severely weakens public health’s ability to measure the benefit from US efforts to curtail uses of these drugs in food-producing animals. Without such assessments, political will in the USA to adopt more targeted surveillance programs or more aggressive measures to curtail usage or institute targeted surveillance programs in the interest of public health also remains elusive.

The closest US analog to DANMAP or MARAN is the National Antimicrobial Resistance Monitoring System (NARMS). NARMS, however, only provides antimicrobial resistance data on bacteria isolated from food producing animals, retail meat products, and human cases of foodborne illness. The NARMS-generated data are not well integrated across the different arms (animal isolates, retail meat isolates, and human clinical isolates are collected independently), and together, these data are not integrated with the summary antimicrobial sales data collected annually by the FDA. This lack of integration severely hampers attempts to examine linkages between antimicrobial use in food animal production and antimicrobial resistance in animals, retail meat, and humans. Denmark’s and The Netherlands’ success in curtailing antimicrobial misuse can be credited to political will, robust systems for collecting and integrating data on antimicrobial consumption and use, and cross-sectoral collaboration. These characteristics have been absent in the USA [5, 89, 105].

## Conclusion

A combination of social, economic, cultural and political factors helps explain why the EU, and especially some of its individual member countries like Denmark and the Netherlands, has enjoyed notable success in reducing antimicrobial consumption in food producing animals [89, 105]. A well-documented set of policies, regulations, and other practices have been promulgated and since shown to be effective. In some EU member-states, implementing multiple strategies has resulted in reductions in antimicrobial consumption of 50% or more — with minimal or no net negative impacts on production costs or profitability, and demonstrable benefits to food safety and public health.

Comparing the overall efficacy and key elements of these European case studies with the experience in the US is illustrative. Many of these key European elements have been incompletely implemented or not attempted at all in the USA, more than a quarter century after Denmark first initiated significant action to curb agricultural antimicrobial use.

The EU has advanced policies to protect the health of its citizens by significantly reducing the use of antimicrobials in animals, especially those important to human medicine. By contrast, US policymakers and government officials have generally not advanced policies to significant reduce antimicrobial use in animals, perhaps due to a lack of political means or will. Possible exceptions include the policies enacted, and implemented with uneven results, in San Francisco, Maryland and California. Gaps in implementation of state and city-based rules in the USA, however, underscore the necessity of much stronger federal leadership in the USA to realize public health protections equivalent to those already enacted or in progress across Europe.

## Supplementary Information

Below is the link to the electronic supplementary material.

Supplementary file1 (DOCX 60 KB)

## Abbreviations

AVMA: American Veterinary Medical Association
CDFA: California Department of Food and Agriculture
DANMAP: Danish Integrated Antimicrobial Resistance Monitoring and Research Program
EMA: European Medicines Agency
ESVAC: European Surveillance of Veterinary Antimicrobial Consumption
FDA: US Food and Drug Administration
IFAP: Industrial food animal production
MARAN: Monitoring of Antimicrobial Resistance and Antimicrobial Use in Animals in Netherlands
MDA: Maryland Department of Agriculture
NARMS: National Antimicrobial Resistance Monitoring System
NRDC: Natural Resources Defense Council
VFD: Veterinary feed directive
