# Supplementary material for: A Review of the Effectiveness of Current US Policies on Antimicrobial Use in Meat and Poultry Production
Source: Curr Environ Health Rep. 2022 Apr 27;9(2):339–54. doi: 10.1007/s40572-022-00351-x (PMC9090690; doi:10.1007/s40572-022-00351-x)
Supplement: Supplementary file 1 — Supplementary file1 (DOCX 60 KB) [file 40572_2022_351_MOESM1_ESM.docx]

**SUPPLEMENT**

**Calculation of U.S. livestock antibiotic consumption on a weight-adjusted (mg/kg) basis**

**Introduction.** Since the European Medicines Agency (EMA) launched its European Surveillance of Veterinary Antimicrobial Consumption (ESVAC) program in September 2009, it has been collecting and reporting country-level data on antibiotics sold for use in food animal production (European Medicines Agency 2011). ESVAC began with the participation of just 8 countries: Denmark, Finland, France, the Netherlands, the United Kingdom, Sweden, Norway, and the Czech Republic. Each provided data on livestock antibiotic sales dating back to 2005. In recent years, 31 European countries have been providing those data.

ESVAC developed and standardized a method for reporting livestock antibiotic sales on a weight-adjusted basis, which entails adjusting the amount of antibiotic (active ingredient) sold by a calculated denominator. EMA calls that denominator the ‘population correction unit”, or PCU; it represents the size of the population of animals (in kilograms) to which antibiotics most likely are administered. Together, the numerator and denominator describe the mg/kg rate of antibiotics used in the production of a particular population of food animals. When sales figures are serving as a proxy for the actual level of antibiotic use on farms, the EMA employs the term “consumption”.

$$Rate of antibiotic consumption=\frac{antibiotic active ingredient sold for use in that sector \left( mg \right)}{calculated livestock weight (PCU,kg)}$$

Both the EMA and the U.S. Food and Drug Administration (FDA) agree that reporting antibiotic sales (or consumption) on a weight-adjusted basis is preferable to reporting antibiotic sales on an unadjusted basis. The FDA, for example, has written that reporting sales on a weight-adjusted basis (United States Food and Drug Administration 2021) would:

“……provide insight into broad shifts in the amount of antimicrobials sold for use in food-producing animals and give the agency a more nuanced view of why sales increase or decrease over time in a manner that is specific to U.S. animal production. Such analysis ***will also support our ongoing efforts to encourage the judicious use of antimicrobials in food-producing animals*** to help ensure the continued availability of safe and effective antimicrobials for animals and humans.” (emphasis added)

In 2017, the FDA proposed a draft method for weight-adjusting its own annual reports of antibiotic sales for food animals (United States Food and Drug Administration 2021). While the EMA has been doing such reporting for more than a decade, the FDA never finalized or implemented its proposal.

The data needed for weight adjustment are available from the FDA, the U.S. Department of Agriculture (USDA), and other sources. Using those data, we have calculated the PCUs for U.S. food animal production for 2016 through 2020, as described below. The accompanying tables summarize the employed data and their sources.

**U.S. calculations**. U.S. federal agencies do no collect annual data on antibiotic use at the farm level. Our calculation of a rate of antibiotic consumption therefore is comprised of the total amount of antibiotics (active ingredient) sold (the numerator), adjusted by the calculated mass of the population of animals to which those antibiotics were most likely administered (the denominator). The calculation necessarily relies on the FDA annual reports detailing sales of antimicrobials for use in food animal production, overall and by animal species.^[[1]](#footnote-2)^

| Cattle antibiotic  sales (mg) | + | Pig antibiotic  sales (mg) | + | Chicken antibiotic  sales (mg) | + | ‘Turkey antibiotic  sales (mg) | = | Total animal  *(cattle + pig + chicken + turkey)*  sales (mg) |
| --- | --- | --- | --- | --- | --- | --- | --- | --- |
|  |  |  |  |  |  |  |  |  |
| Cattle  PCUs (kg) | **+** | Pig  PCUs (kg) | **+** | Chicken PCUs (kg) | **+** | Turkey  PCUs (kg) | = | Total PCUs (kg) |

We used the EMA’s well-established methodology to calculate PCUs for these four major types of food animals produced in the United States, using public USDA data for 2020 on the annual slaughter or inventories of animals at various life stages. To reiterate, the PCUs represent the size in kilograms of the population of animals to which sold antibiotics would most likely have been administered.

Supplemental Table 1 shows how antibiotic sales and PCUs are combined to describe the antibiotic consumption intensity for 2020 for U.S. food animal production overall, which is 170.8 mg/kg, as well as for the four major food animal sectors: cattle, pigs, chickens and turkeys.

**Supplemental Table 1. Rate of antibiotic consumption in U.S. food animal production, 2020**

| Animal Antibiotic Sales (kg) | **Cattle** | **Pigs** | **Chickens** | **Turkeys** | **Total** |
| --- | --- | --- | --- | --- | --- |
|  | 2,449,441 | 2,451,382 | 141,793 | 690.841 | 6,002,056 |
| Calculated weight, or PCUs (kg) | 15,190,059,470 | 9,149,283,235 | 9,339,474,289 | 1,449,519,500 | 35,136,336,494 |
| Rate of antibiotic consumption: mg of antibiotic / kg-livestock | **161.3** | **267.9** | **15.2**  **5** | **476.6** | **170.8** |

Supplemental Table 2 presents the data collected from U.S. agencies for calculation of PCUs by animal sector for 2020. Summing the calculated PCUs for the four major animal species – cattle, pigs, broiler chickens and turkeys – gives the overall PCUs calculated for the United States for 2020, which is 35.1B kilograms.

**Supplemental Table 2. Calculation of PCUs for the United States, 2020**

|  | **Number** (in thousands) | **Avg. Weight (kg)** | **Calculated weight, PCUs or kilograms** |
| --- | --- | --- | --- |
| **Cattle** |  |  |  |
| Number of slaughtered cows | 6,331.4  ^a^ | 425 | 2,690,845,000 |
| Number of slaughtered heifers | 9.335.3 ^a^ | 200 | 1,889,060,000 |
| Number of slaughtered bulls | 518.3  ^a^ | 425 | 220.277.500 |
| Number of slaughtered calves | 446.8  ^a^ | 140 | 62,552,000 |
| Number of slaughtered steers | 15,856.2  ^a^ | 425 | 6,378,885,000 |
| Number of imported cattle for slaughter (-) | 532.5  ^b^ | -425 | (226,298,050) |
| Number of Imported cattle for fattening (+) | 1,571.0^b^ | -140 | (219,934,820) |
| Number of exported cattle for fattening (-) | 319.8  ^b^ | 140 | 44,772,840 |
| Number of livestock dairy cows | 9,388.0  ^c^ | 425 | 3,989,900,000 |
| ***Semi-total, cattle*** |  |  | ***15,190,059.470*** |
| **Pigs** |  |  |  |
| Number of slaughtered pigs | 131,563,000^a^ | 65 | 8,551,595,000 |
| Imported fattening pigs | 4,436.5^d^ | -25 | (110,913,550) |
| Exported fattening pigs | 28.7^e^ | 25 | 717,625 |
| Imported slaughter pigs | 802.9^f^ | -65 | (52,186,615) |
| Exported slaughter pigs | 10.9^g^ | 65 | 710,775 |
| Livestock sows (sows farrowed) | 3,164.0^h^ | 240 | 759,360,000 |
| ***Semi-total, pigs*** |  |  |  |
| **Poultry** |  |  |  |
| Slaughtered chickens | 9,346,660,000^i^ | 1 | 9,346,660,000 |
| Imported chickens | 33,516 ^j^ | -1 | (33,516) |
| Exported chickens | 847,805 ^j^ | 1 | 847,805 |
| ***Semi-total, chicken*** |  |  | ***9,347,474,289*** |
| ***Semi-total, slaughtered turkeys*** | 223,003.0 ^k^ | 6.5 | ***1,449,519,500*** |
| **Total** |  |  |  |

190,059.5

a) USDA, National Agricultural Statistics Service (NASS). Livestock Slaughter, 2020 Summary, dated April 22, 2021. Page 17, Table entitled "Federally Inspected Slaughter and Percent by Classification and Month – United States: 2020 and 2019 Total". Retrieved at thttps:// downloads.usda.library.cornell.edu/
usda-esmis/files/ r207tp32d/ sj139x554/ 7w62g4561/lsan0421.pdf;

b) Economic Research Service webpage. Cattle: Annual and cumulative year-to-date U.S. trade - All years and countries, "Cattle imports, cattle and calves for feeding", Cattle imports for slaughter", and “Cattle exports, total”. Retrieved from https://www.ers.usda.gov/ data-products/livestock-and-meat-international-trade-data/;

c) USDA, National Agricultural Statistics Service (NASS) Quick Stats, https://quickstats.nass.usda.gov/ results/ E0CFD1BD-790D-341C-853F-A5727C3EC03D;

d) ERS webpage, Hogs: Annual and cumulative year-to-date U.S. trade - All years and countries, "Hog imports, less than 7kg" + "Hog imports, 7 - less than 23 kg", + "Hog imports, 23 to less than 50 kg" retrieved from https://www.ers.usda.gov/ data-products/livestock-and-meat-international-trade-data/;

e) ERS webpage, Livestock and Meat International Trade Data, Annual and Cumulative Year-to-Date U.S. Livestock and Meat Trade by Country, Hogs: Annual and cumulative year-to-date U.S. trade - All years and countries, "Hog exports, less than 50 kg", retrieved from https:/www.ers.usda.gov/data-products/livestock-and-meat-international-trade-data/;

f) ERS webpage, Hogs: Annual and cumulative year-to-date U.S. trade - All years and countries, "Hog imports, 50 kg or more for immediate slaughter", retrieved from https://www.ers.usda.gov/data-products/livestock-and-meat-international-trade-data/

g) ERS webpage, Livestock and Meat International Trade Data, Annual and Cumulative Year-to-Date U.S. Livestock and Meat Trade by Country, Hogs: Annual and cumulative year-to-date U.S. trade - All years and countries, "Hog exports, 50 kg or more", retrieved from https://www.ers.usda.gov/data-products/livestock-and-meat-international-trade-data/;

h) USDA, NASS, United States and Canadian Hogs, Table 3: "Hogs and Pigs Inventory, Sows Farrowed, and Pig Crop – United States: 2015-2020. March 2021, retrieved at https://downloads.usda.library.cornell.edu/usda-esmis/files/7h149p85x/ dr26zr477/kk91gd87k/usch0321.pdf;

i) USDA, NASS, Poultry Slaughter: Annual Summary, "Poultry Slaughtered, Total Live Weight, and Average Live Weight by Type and Month – United States: 2020 and 2019 totals, page 5, accessed March 8, 2021 at https://www.nass.usda.gov/ Publications/TodaysReports/reports/psla0120.pdf;

j) International Trade Centre, ITC Trade Map. Search for United States imports from the world of Product Code #010511, "Live fowls of the species Gallus domesticus weighing > 185, imported by the US", (which is chicken), access via https://www.trademap.org/Index.aspx;

k) USDA, NASS, Poultry Slaughter: Annual Summary, "Poultry Slaughtered, Total Live https://www.nass.usda.gov/Publications/Todays_Reports/reports/psla0120.pdf.

**REFERENCES**

European Medicines Agency. 2011. Trends in the sales of veterinary antimicrobial agents in nine european countries, reporting period 2005-2009. Available: <https://www.ema.europa.eu/en/documents/report/trends-sales-veterinary-antimicrobial-agents-nine-european-countries_en.pdf> 29 September 2021].

United States Food and Drug Administration. 2021. Fda’s proposed method for adjusting data on antimicrobials sold or distributed for use in food-producing animals, using a biomass denominator Available: <https://www.fda.gov/files/animal%20&%20veterinary/published/FDA%E2%80%99s-Proposed-Method-for-Adjusting-Data-on-Antimicrobials-Sold-or-Distributed-for-Use-in-Food-Producing-Animals-Using-a-Biomass-Denominator--Technical-Paper.pdf> 29 September 2021].

1. United States Food and Drug Administration. 2021. 2020 summary report on antimicrobials sold or distributed for use in food-producing animals. Available: https://www.fda.gov/media/154820/download 24 February 2022. [↑](#footnote-ref-2)
